# Atomic Layer Deposition of Ultrathin ZnO Films for Hybrid Window Layers for Cu(In_x_,Ga_1−x_)Se_2_ Solar Cells

**DOI:** 10.3390/nano11112779

**Published:** 2021-10-20

**Authors:** Jaebaek Lee, Dong-Hwan Jeon, Dae-Kue Hwang, Kee-Jeong Yang, Jin-Kyu Kang, Shi-Joon Sung, Hyunwoong Park, Dae-Hwan Kim

**Affiliations:** 1 Research Center for Thin Film Solar Cells, Daegu-Gyeongbuk Institute of Science and Technology (DGIST), Daegu 42988, Korea; tglee@dgist.ac.kr (J.L.); dhjeon@dgist.ac.kr (D.-H.J.); dkhwang@dgist.ac.kr (D.-K.H.); kjyang@dgist.ac.kr (K.-J.Y.); apollon@dgist.ac.kr (J.-K.K.); 2 Division of Energy Technology, Daegu-Gyeongbuk Institute of Science and Technology (DGIST), Daegu 42988, Korea; 3 School of Energy Engineering, Kyungpook National University, Daegu 41566, Korea

**Keywords:** ZnO, atomic layer deposition, ultrathin, window layer, CIGS, solar cells

## Abstract

The efficiency of thin-film chalcogenide solar cells is dependent on their window layer thickness. However, the application of an ultrathin window layer is difficult because of the limited capability of the deposition process. This paper reports the use of atomic layer deposition (ALD) processes for fabrication of thin window layers for Cu(In_x_,Ga_1−x_)Se_2_ (CIGS) thin-film solar cells, replacing conventional sputtering techniques. We fabricated a viable ultrathin 12 nm window layer on a CdS buffer layer from the uniform conformal coating provided by ALD. CIGS solar cells with an ALD ZnO window layer exhibited superior photovoltaic performances to those of cells with a sputtered intrinsic ZnO (i-ZnO) window layer. The short-circuit current of the former solar cells improved with the reduction in light loss caused by using a thinner ZnO window layer with a wider band gap. Ultrathin uniform A-ZnO window layers also proved more effective than sputtered i-ZnO layers at improving the open-circuit voltage of the CIGS solar cells, because of the additional buffering effect caused by their semiconducting nature. In addition, because of the precise control of the material structure provided by ALD, CIGS solar cells with A-ZnO window layers exhibited a narrow deviation of photovoltaic properties, advantageous for large-scale mass production purposes.

## 1. Introduction

Thin-film chalcogenide solar cells such as Cu(In_x_,Ga_1−x_)Se_2_ (CIGS), Cu_2_ZnSnSe_4_ (CZTSe), and SnS typically require an intrinsic ZnO (i-ZnO) window layer, deposited between their CdS buffer layer and transparent conducting oxide (TCO) layers, to improve device performance without increasing light absorption loss [1,2,3,4]. This i-ZnO window layer effectively blocks the short-circuit pathways through the voids in the CdS buffer layer, and consequently enhances the shunt resistance (R_sh_), fill factor (FF), and open-circuit voltage (V_OC_) of the solar cells. In addition, the i-ZnO window layer protects the CdS buffer layer during the subsequent deposition of a TCO layer [5,6,7,8].

Sputtering processes are typically used for deposition of the window layers of CIGS thin-film solar cells because of their fast deposition rate, and the strong adhesion of i-ZnO to CdS [9,10]. However, as such techniques are incapable of depositing ultrathin uniform ZnO films, only a narrow range of process parameters are suitable for the sputtering process of i-ZnO window layers. In general, the thickness of a ZnO window layer must be greater than 50 nm to cover the rough CdS/CIGS surface. The thickness of the ZnO window layer is closely related to its parasitic absorption loss, which induces a decrease in the short-circuit current (J_SC_) of CIGS solar cells. Therefore, a reduction in the thickness of the ZnO window layer is required to achieve a higher value of J_SC_. However, the surface coverage of a thin sputtered i-ZnO layer on a CdS buffer layer is insufficient to prevent current leakage from CIGS solar cells. In addition, the severe sputter process conditions required for deposition of i-ZnO can cause critical damage to the underlying CdS buffer layer [11,12].

To overcome the drawbacks of sputter coating, atomic layer deposition (ALD) processes have been proposed for fabrication of the ZnO window layers in CIGS thin-film solar cells. Although the ALD technique is a well-established deposition method for precise fabrication of ultrathin films [13,14,15], few studies have reported on ALD of ZnO window layers for thin-film chalcogenide solar cells [5,16,17]. Such reports that exist have focused on the material properties of atomic-layer-deposited ZnO (A-ZnO). In these studies, the thickness of the A-ZnO window layer remained over 50 nm, and the efficiencies of the corresponding CIGS thin-film solar cells were below 13%. Hence, the strengths of ALD for fabrication of ZnO window layers in chalcogenide solar cells were not practically utilized. 

In this work, we discuss the fabrication of ultrathin uniform A-ZnO window layers for high-efficiency CIGS solar cells. To maximize the strengths of the ALD technique, we focus on deposition of thin films with thicknesses between 12 and 23 nm. ALD enables conformal and uniform coating of ZnO on the rough surface of a CdS buffer layer. Hence, ultrathin A-ZnO window layers provide sufficient passivation and protection to the CdS buffer layer, enabling lower thicknesses of ZnO to be used in CIGS solar cells, compared to those required with conventional sputtering. In addition, reducing the thickness of the ZnO window layer is advantageous for the formation of a built-in field over the ZnO window layer, as the spreading of the space charge region can be depressed [18]. As well as reducing its thickness, using ALD instead of sputtering can increase the band gap of the window layer [19], which is advantageous for reducing the light absorption loss of CIGS solar cells in the short wavelength range. This reduced loss can subsequently induce an improvement in the J_SC_ of CIGS solar cells.

Unlike sputtered i-ZnO, which is highly resistive, A-ZnO has electrical properties characteristic of a weak n-type semiconductor [20], making it beneficial for improving the V_OC_ of CIGS solar cells. Carrier transport through the void regions of the buffer layer is easier with an A-ZnO window layer than with a sputtered i-ZnO window layer, as the former material infiltrates the CdS, which increases the V_OC_ of CIGS solar cells. Weak n-type semiconducting A-ZnO window layers can thus act as secondary buffer layers in CIGS solar cells. In contrast, ultrathin ZnO layers deposited by sputtering cannot play this role, as the material does not fill the voids in the CdS buffer layer.

The discussion above demonstrates the potential of A-ZnO window layers in high-efficiency CIGS solar cells, by explaining how the ALD technique can improve both the V_OC_ and J_SC_ of CIGS solar cells. In addition, because of the uniform and conformal coating of ALD, CIGS solar cells with an A-ZnO window layer can be expected to exhibit a narrow statistical deviation of photovoltaic parameters, useful for large-scale mass production of CIGS solar cells. The proposed A-ZnO window layer can also be applied to other chalcogenide solar cells, regardless of their interfacial structures.

## 2. Materials and Methods 

### 2.1. Device Fabrication

The solar cells investigated in this study consisted of a soda lime glass (SLG) substrate, a 500 nm thick Mo back-contact layer, an approximately 2.5 μm thick CIGS absorber layer, a 60 nm thick CdS buffer layer, i-ZnO or ZnO window layers, a 300 nm thick Al-doped ZnO (AZO) transparent conducting oxide layer, and a 1 μm thick Al collection grid. The Mo layer was deposited on the SLG substrate via DC magnetron sputtering using a Mo target with a purity of 99.99%. Following this, the CIGS absorber was deposited on the back-contact layer by thermal co-evaporation from elemental sources. Here, evaporation was completed in a multi-stage process. First, In, Ga, and Se were deposited on the substrate at a temperature of 430 °C. Following this, Cu and Se were evaporated on the substrate at a temperature of 650 °C, until the absorber became copper-rich. The process was completed by evaporation of In, Ga, and Se, to make the absorber copper-poor again. Next, the CdS layer was deposited onto the device using a wet-chemical process, followed by window layer deposition using sputtering processes or ALD (see Section 2.2), as appropriate. Finally, the 300 nm thick AZO front-contact layer was deposited by radio frequency (RF) sputtering, followed by thermal evaporation of 1 μm Al grids. Solar cells with a total area of 0.5535 cm^2^ were defined by mechanical scribing.

### 2.2. ALD Process

A-ZnO window layers were obtained through ALD of diethyl zinc ((C_2_H_5_)_2_Zn; UP Chemical Co., Ltd., Gyeonggi-do, Korea) and deionized water on the surface of the CdS buffer layer. Deposition was completed using a showerhead-type ALD system (CN1 Co., Ltd. Gyeonggi-do, Korea). ATOMIC PREMIUM), with each ALD cycle performed at a temperature of 120 °C. The thickness of the thin films was modified by varying the number of ALD cycles, and 100 ALD cycles performed at 120 °C obtained a final ZnO thickness of 23 nm. The sub-ALD cycle consisted of 0.5 s of dosing with diethyl zinc precursor, 15 s of purging with N_2_, 0.5 s of dosing with H_2_O, and a further 15 s of purging with N_2_. 

### 2.3. Device Characterization

The solar cell samples were characterized using a combination of field-emission transmission electron microscopy (FE-TEM), atomic force microscopy (AFM), solar simulator, and external quantum efficiency (EQE) measurement. The cross-sectional morphologies of the CIGS solar cells were investigated using an FE-TEM system (HF-3300, Hitachi, Saitama, Japan) with a focused ion beam (FIB) instrument (NB5000, Hitachi, Saitama, Japan). The surface morphologies of A-ZnO and sputtered i-ZnO were investigated using a Park NX10 AFM instrument (Park systems, Gyeonggi-do, Korea). The current-voltage characteristics of the solar cells under a simulated air mass 1.5 global (AM 1.5 G) spectrum were measured at an illumination of 100 mW cm^−2^ (1 sun) using a solar simulator (model 94022A, Newport Co., Irvine, CA, USA). The EQE spectra were measured using a QuantX-300 measuring kit (Newport Co., Irvine, CA, USA).

## 3. Results and Discussion 

### 3.1. Formation of A-ZnO Window Layers on a CdS/CIGS Interface

**Figure 1:**
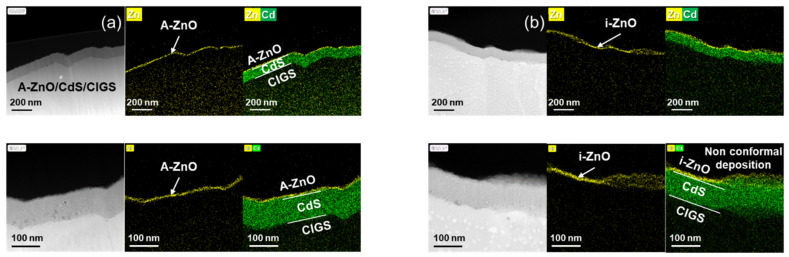
Cross-sectional scanning transmission electron microscopy (STEM) high-angle annular dark-field imaging (HAADF) and energy-dispersive X-ray spectroscopy (EDS) images of (**a**) atomic-layer-deposited ZnO (A-ZnO) on a CdS/CIGS surface and (**b**) sputtered intrinsic ZnO (i-ZnO) on a CdS/CIGS surface.

Exploiting the advantages of ALD with respect to conventional sputtering depends on the precise conformal deposition of the ultrathin material. Hence, cross-sectional STEM-HADDF and EDS images of the interface of the CdS buffer layer and the CIGS absorber layer were acquired to confirm the formation of a window layer (Figure 1). The differing nature of ALD and sputtering processes was analyzed based on the morphology of ZnO thin films with a nominal thickness of 12 nm. In the case of ALD, a uniform ZnO thin film was deposited on the rough surface of the CdS buffer layer. In contrast, there were large variations in the thickness of the sputtered i-ZnO thin film, including the presence of regions on the CdS layer where no material was deposited. This non-uniform window layer confirms the inability of the sputtering process to deposit ultrathin conformal coatings, explaining why the minimum window layer thickness previously reported has been 50 nm.

**Figure 2:**
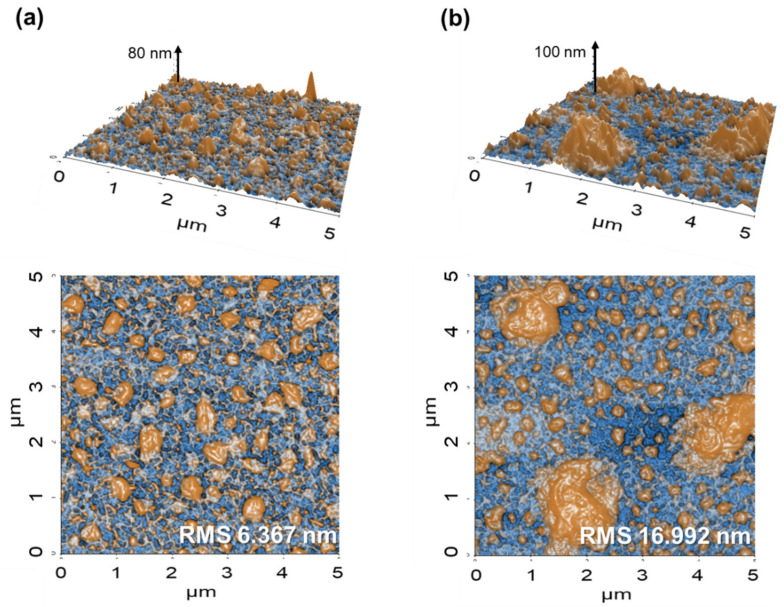
Atomic force microscopy (AFM) images of (**a**) A-ZnO on SLG, and (**b**) sputtered i-ZnO on SLG.

We conducted AFM analysis of 46 nm thick ZnO thin films on SLG, for further comparison of the difference between ALD and sputtering processes (Figure 2). Again, the A-ZnO thin film exhibited a uniform morphology, with a regular distribution of small protrusions. In contrast, the sputtered i-ZnO thin film exhibited a non-uniform surface morphology, with large protrusions occupying the spaces between the small protrusions. The different surface morphologies of the atomic-layer-deposited and sputtered ZnO thin films are also reflected in their root-mean-square roughness (R_q_), with values of 6.367 nm obtained for the A-ZnO film, and 16.992 nm for the sputtered i-ZnO film. This result confirms that, by exploiting the unique characteristics of ALD, an ultrathin uniform ZnO window layer can be successfully deposited on a rough CdS buffer layer.

### 3.2. Performance of A-ZnO Window Layers in CIGS Solar Cells

**Figure 3:**
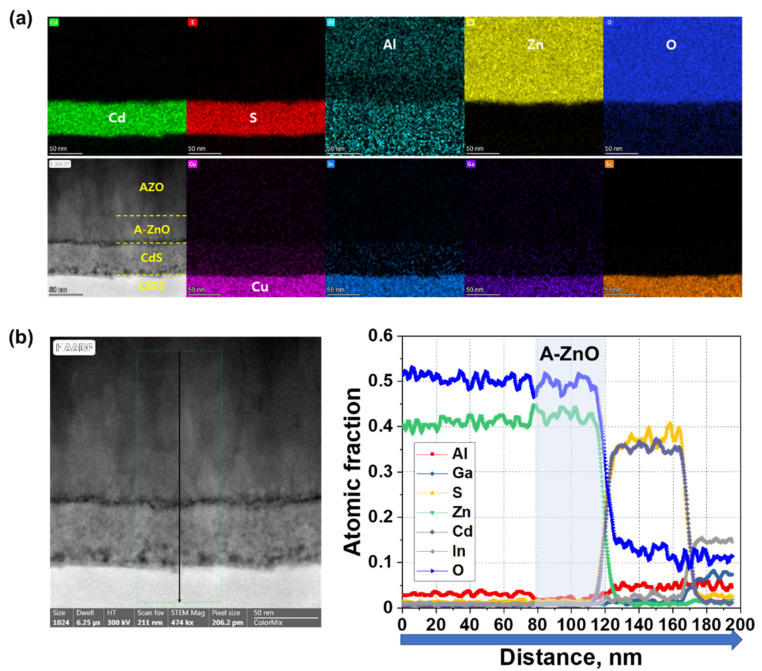
(**a**) Cross-sectional TEM energy dispersive spectroscopy (EDS) mapping images and (**b**) EDS line scan of atomic fraction of elements in the AZO/A-ZnO/CdS/CIGS/Mo/SLG structure of a complete solar cell.

We acquired cross-sectional TEM images of complete CIGS solar cell devices (Figure 3) to confirm that the window layer remained uniform following deposition of the front contact layers, as this uniformity is essential for improved device performance. The energy dispersive spectroscopy (EDS) mapping images of the CIGS solar cells (Figure 3a) depict a uniform A-ZnO window layer between the CdS buffer layer and the AZO TCO layer. However, it is difficult to distinguish the A-ZnO window layer from the AZO TCO layer because of signal noise from elemental Al. Hence, we performed an EDS line scan to verify the formation of the A-ZnO window layer. These measurements highlighted a region without elemental Al between the CdS buffer layer (120–170 nm) and the AZO TCO layer (0–80 nm) (Figure 3b), indicating the formation of a separated A-ZnO window layer inside the CIGS solar cell device. No signals were observed in this region during EDS mapping of elemental Al at different locations in the CIGS solar cell device (Supplementary Figure S1), confirming our hypothesis. 

**Figure 4:**
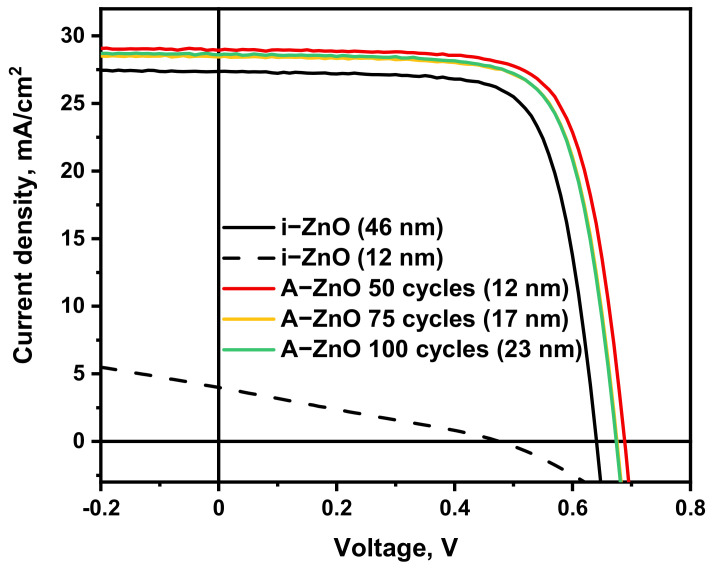
Characteristic current-voltage curves of CIGS solar cells with sputtered i-ZnO and A-ZnO window layers with different thicknesses.

The photovoltaic properties of CIGS solar cells with A-ZnO (with thicknesses of 12–23 nm) were investigated to evaluate the performances of the thin films as window layers. CIGS solar cell devices with sputtered i-ZnO window layers with thicknesses of 12 and 46 nm were also prepared, for comparison. Figure 4 and Table 1 summarize the photovoltaic characteristics of both sets of CIGS solar cell devices.

Our characterization indicates that CIGS solar cells with A-ZnO window layers exhibited higher efficiency than cells with sputtered i-ZnO window layers, regardless of the thickness of the A-ZnO window layer. This improved efficiency originates from the higher V_OC_ and J_SC_ of the former devices, indicating that ultrathin A-ZnO films are suitable for use as the window layer of CIGS solar cells. Although the device with the 12 nm A-ZnO window layer exhibited the best photovoltaic properties, overall, modifying the thickness of the A-ZnO window layers had little effect on the photovoltaic properties of the CIGS solar cells. In contrast, the device with a 12 nm thick sputtered i-ZnO window layer exhibited a poor efficiency of under 2%. This inferior photovoltaic performance can be attributed to the non-uniform formation of the sputtered ultrathin i-ZnO window layer on the CdS buffer layer, which was depicted in Figure 1. The non-uniform coverage of the CdS buffer layer by the sputtered i-ZnO window layer is insufficient to protect the CdS buffer layer from damage during the AZO TCO sputtering process, which deteriorates the photovoltaic performance of the CIGS solar cells. This result indicates that ALD is necessary for the application of ultrathin ZnO window layers in CIGS solar cells.

**Figure 5:**
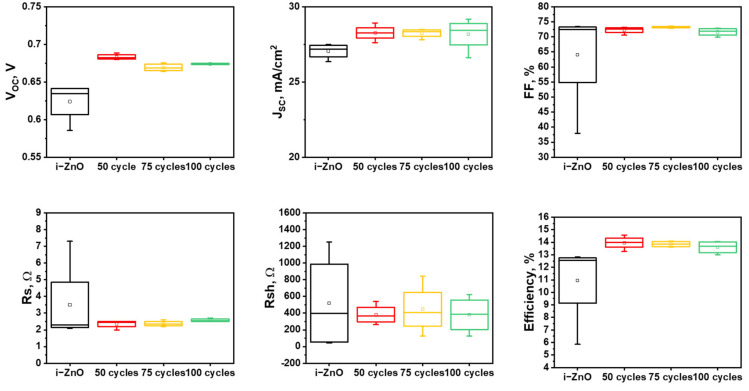
Photovoltaic parameters of CIGS solar cells with i-ZnO and A-ZnO window layers of different thickness.

Figure 1 and Figure 2 highlighted that a distinguishing characteristic of ALD is uniform formation of ultrathin conformal ZnO window layers. For additional evaluation of the merit of this characteristic, we conducted a statistical analysis of the photovoltaic parameters of CIGS solar cells with both A-ZnO and sputtered i-ZnO window layers (Figure 5). With the exception of the short-circuit current (J_SC_), CIGS solar cells with a sputtered ZnO window layer exhibited large deviations in photovoltaic parameters compared to devices with A-ZnO window layers, with the FF, series resistance (R_s_), and efficiency exhibiting particularly large variances. These large deviations can be attributed to the non-uniformity of the sputtered i-ZnO window layer. The large variation in the thickness of the 12 nm sputtered i-ZnO film hinders the ability of the window layer to prevent current leakage. The large deviation in FF is thus closely related to the large deviation in the efficiency of CIGS solar cells. In contrast, CIGS solar cells with A-ZnO thin films showed very narrow deviations in V_OC_, FF, and R_s_, indicating that, because of their uniform and conformal deposition on the CdS buffer layer, they are more effective as window layers than sputtered i-ZnO films. The small deviation in the photovoltaic parameters of CIGS solar cells with A-ZnO window layers is also meaningful for the purposes of scalable manufacture, as it suggests the potential for rapid fabrication of devices with repeatable performance.

### 3.3. Effect of A-ZnO Window Layers on Photovoltaic Parameters

The analysis of the photovoltaic properties of CIGS solar cells above demonstrates that ultrathin A-ZnO films can act as the window layer in such devices, without the deterioration of their performance. The unique structural properties of the A-ZnO thin films enable them to provide sufficient protection to the CdS buffer layer during deposition of the front-contact layer, despite their reduced thickness. In addition, both CIGS solar cells with A-ZnO window layers and the device with the 46 nm sputtered i-ZnO window layer exhibited similar values of R_sh_, indicating that an ultrathin A-ZnO window layer suitably prevents current leakage. Since ALD creates a uniform conformal ZnO window layer using the ALD process, the deviation of R_sh_ with devices with such coatings is smaller than that of devices with a sputtered i-ZnO window layer. This explains why the photovoltaic properties of CIGS solar cells with A-ZnO films are similar, regardless of the thickness of the window layer. The narrow distribution of the V_OC_ of the CIGS solar cells with A-ZnO window layers can thus be attributed to the narrow deviation of R_sh_. 

**Figure 6:**
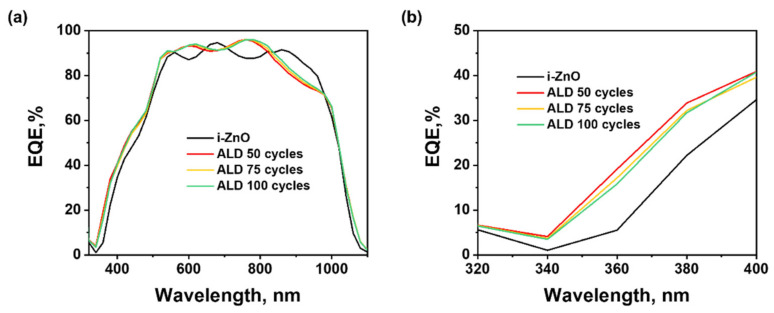
External quantum efficiency (EQE) of CIGS solar cells with i-ZnO and A-ZnO window layers with different thickness in (**a**) the full wavelength range (300–1100 nm), and the (**b**) short-wavelength region (~320–400 nm).

The improved photovoltaic performance of CIGS solar cells with A-ZnO window layers results from the improvement of J_SC_ and V_OC_. The origin of these improved photovoltaic characteristics can be explained by considering how the change in the physical characteristics of A-ZnO films modify their optical and electrical properties with respect to those of sputtered i-ZnO films. It has previously been demonstrated that the optical band gap of ZnO thin films is closely related to the conditions used in chemical vapor deposition (CVD) [20]. Accordingly, the differing processes used in deposition of the window layers affected their band gaps, with A-ZnO films exhibiting a wider band gap (3.33 eV) than the sputtered i-ZnO film (3.21 eV) (Supplementary Figure S2). In addition, in this study, the thickness of the A-ZnO window layer was less than half that of the sputtered i-ZnO window layer. The combination of the wider band gap and smaller thickness of the A-ZnO window layer are advantageous for minimizing its light absorption loss, and improving the optical performance of CIGS solar cells. To verify this assertion, we obtained the EQE spectra of both CIGS solar cells with A-ZnO window layers and the CIGS solar cells with a 50 nm sputtered i-ZnO window layer (Figure 6). In the short wavelength range under 550 nm, CIGS solar cells with A-ZnO window layers exhibited a higher EQE, confirming the effect of their wider band gap. In addition, the EQE decreased with increasing ALD cycles, confirming that layer thickness affects light absorption loss. Although the spectra of A-ZnO and sputtered i-ZnO films varied differently at wavelengths over 550 nm, the average EQE of both types of thin film was similar in this range, indicating that ALD is most effective in improving the light absorption loss of CIGS solar cells in the short-wavelength region. The higher values of EQE for wavelengths under 550 nm thus contribute to the increased J_SC_ of CIGS solar cells with A-ZnO window layers. 

Similarly, the modified electrical properties of the A-ZnO thin films can explain the improvement in the V_OC_ of CIGS solar cells with such coatings. A comparison of the electrical properties of the A-ZnO and sputtered i-ZnO thin films from the Hall measurement is included in Table 2. The A-ZnO films exhibited higher conductivities, lower resistivities, and higher carrier concentrations than the sputtered i-ZnO film, attributes that are consistent with their well-known similarity to weak n-type semiconductors [20]. The higher V_OC_ of CIGS solar cells with A-ZnO window layers can thus be explained by the additional buffering effect provided by the weak n-type semiconductor layer. By infiltrating the void region of the CdS buffer layer, the A-ZnO thin film can function as a secondary buffer layer, facilitating smooth carrier transport. Sputtered i-ZnO window layers are unable to function in this way, explaining the improved V_OC_ observed with A-ZnO thin films.

## 4. Conclusions

In this study, we substituted the conventionally sputtered i-ZnO window layers in CIGS solar cells with A-ZnO thin films. Our characterization highlighted that devices using the latter material exhibited superior photovoltaic performance to those using the former. We also demonstrated that ultrathin A-ZnO films (~12 nm) acted suitably well as the window layer of CIGS solar cells, because the ALD process enabled uniform conformal coating of the CdS buffer layer. The CIGS solar cell using an ultrathin A-ZnO window layer showed higher values of efficiency (14.578%), V_OC_ (0.68862 V), J_SC_ (28.9264 mAcm^−^^2^), and FF (73.1868%) than the CIGS solar cell using a sputtered i-ZnO. The improved performance of CIGS solar cells with A-ZnO window layers can be explained by enhancements to their J_SC_ (from 27.3669 to 28.9264 mAcm^−2^) and V_OC_ (from 0.64068 to 0.68862 V). These enhancements result from the modified optical and electrical properties of the window layer, caused by the ALD process’ creation of a ZnO film with a more uniform structure. The enhancement of the J_SC_ of these CIGS solar cells was caused by their improved light absorption loss, resulting from the fabrication of a thin film with a wider band gap, and the ability to employ thinner ZnO films in the window layer using ALD. In addition, we observed that the electrical properties of A-ZnO thin films are characteristic of weak n-type semiconductors, enabling the window layers to facilitate smooth carrier transport through the void region of the CdS buffer layer. By infiltrating the CdS layer, the A-ZnO thin film functioned as a secondary buffer layer in CIGS solar cells, enabling the improvement in V_OC_. In addition to the improvement in the photovoltaic performance of the CIGS solar cells with A-ZnO window layers, we observed smaller deviations in the photovoltaic parameters of individual solar cells because of the precise control of window layer structure enabled by our use of ALD. Hence, as well as providing a promising substitute to conventionally sputtered ZnO window layers, the proposed ALD process can also facilitate the large-scale mass production of CIGS solar cells.

## Figures and Tables

**Table 1:** Photovoltaic parameters of CIGS solar cells with sputtered i-ZnO and A-ZnO window layers of different thickness.

	Thickness (nm)	V_OC_ (V)	J_SC_ (mA/cm^2^)	FF (%)	Efficiency (%)	R_s_ (Ω)	R_sh_ (Ω)
i-ZnO (Ref.)	46	0.64068	27.3669	73.0578	12.809	2.4	723.6
i-ZnO	12	0.47154	14.0397	25.9519	1.736	16.4	66.8
A-ZnO (50 cycles)	12	0.68862	28.9264	73.1868	14.578	2.0	539.3
A-ZnO (75 cycles)	17	0.67545	28.4352	73.2842	14.075	2.6	365.8
A-ZnO (100 cycles)	23	0.67429	28.5909	72.9267	14.059	2.6	490.5

**Table 2:** Electrical properties of sputtered i-ZnO and A-ZnO thin films.

	Mobility (cm^2^/V⋅s)	Conductivity (1/Ω⋅cm)	Resistivity (Ω⋅cm)	Carrier Concentration (cm^−3^)
Sputtered i-ZnO	14.77	7.684 × 10^−6^	1.301 × 10^5^	−3.247 × 10^12^
A-ZnO	12.99	14.59	6.852 × 10^−2^	−7.015 × 10^18^

## Data Availability

The data presented in this study are available on request from the corresponding author.

## Supplementary Materials

The following are available online at https://www.mdpi.com/article/10.3390/nano11112779/s1, Figure S1: EDS data of an aluminum (Al) element in different positions of a CIGS solar cell device, Figure S2: Transmittance and optical band gap properties of the i-ZnO and A-ZnO: (a) transmittance spectra of i-ZnO and A-ZnO, (b) optical band gap of i-ZnO, and (c) optical band gap of A-ZnO.
