# Supplementary material for: Atomic Layer Deposition of Ultrathin ZnO Films for Hybrid Window Layers for Cu(Inx,Ga1−x)Se2 Solar Cells
Source: Nanomaterials (Basel). 2021 Oct 20;11(11):2779. doi: 10.3390/nano11112779 (PMC8619686; doi:10.3390/nano11112779)
Supplement: Supplementary file 1 [file nanomaterials-11-02779-s001.zip › nanomaterials-1414130-supplementary.pdf]

Supplementary Materials

for

Atomic Layer Deposition of Ultrathin ZnO Films for Hybrid Window Layers for $Cu(In_x,Ga_{1-x})Se_2$ Solar Cells

Jaebaek Lee [1,2,3], Dong-Hwan Jeon [1,2], Dae-Kue Hwang [1,2], Kee-Jeong Yang [1,2], Jin-Kyu Kang [1,2], Shi-Joon Sung [1,2,*], Hyunwoong Park [3,*] and Dae-Hwan Kim [1,2,*]

1. Research Center for Thin Film Solar Cells, Daegu-Gyeongbuk Institute of Science and Technology (DGIST), Daegu 42988, Korea
2. Division of Energy Technology, Daegu-Gyeongbuk Institute of Science and Technology (DGIST), Daegu 42988, Korea
3. School of Energy Engineering, Kyungpook National University, Daegu 41566, Korea

* Correspondence: sjsung@dgist.ac.kr (S.-J.S.); hwp@knu.ac.kr (H.P.); monolith@dgist.ac.kr (D.-H.K.)

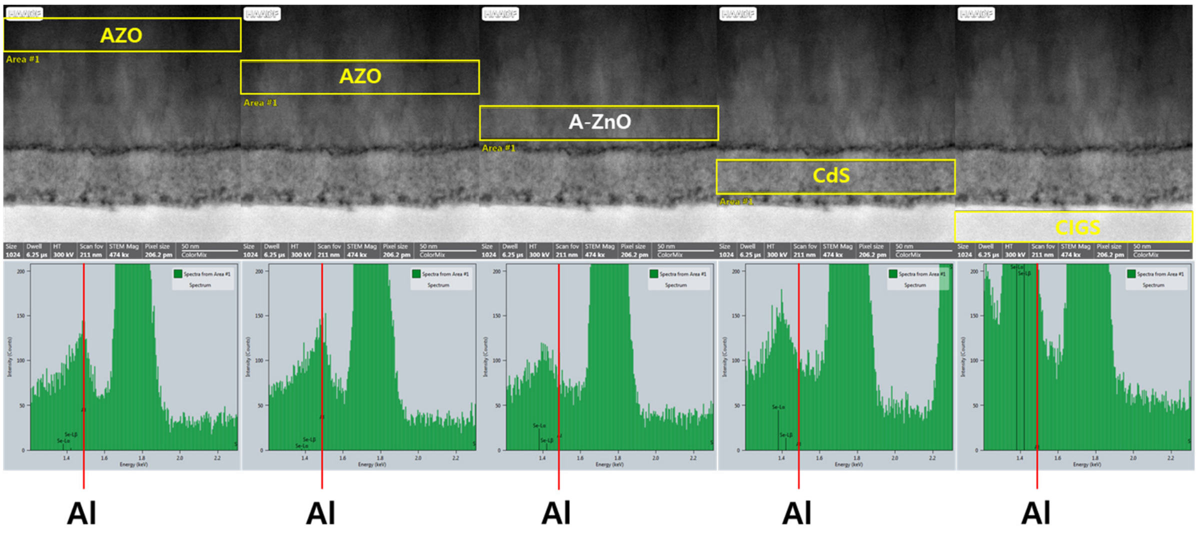

Figure S1. EDS data of an aluminum (Al) element in different positions of a CIGS solar cell device.

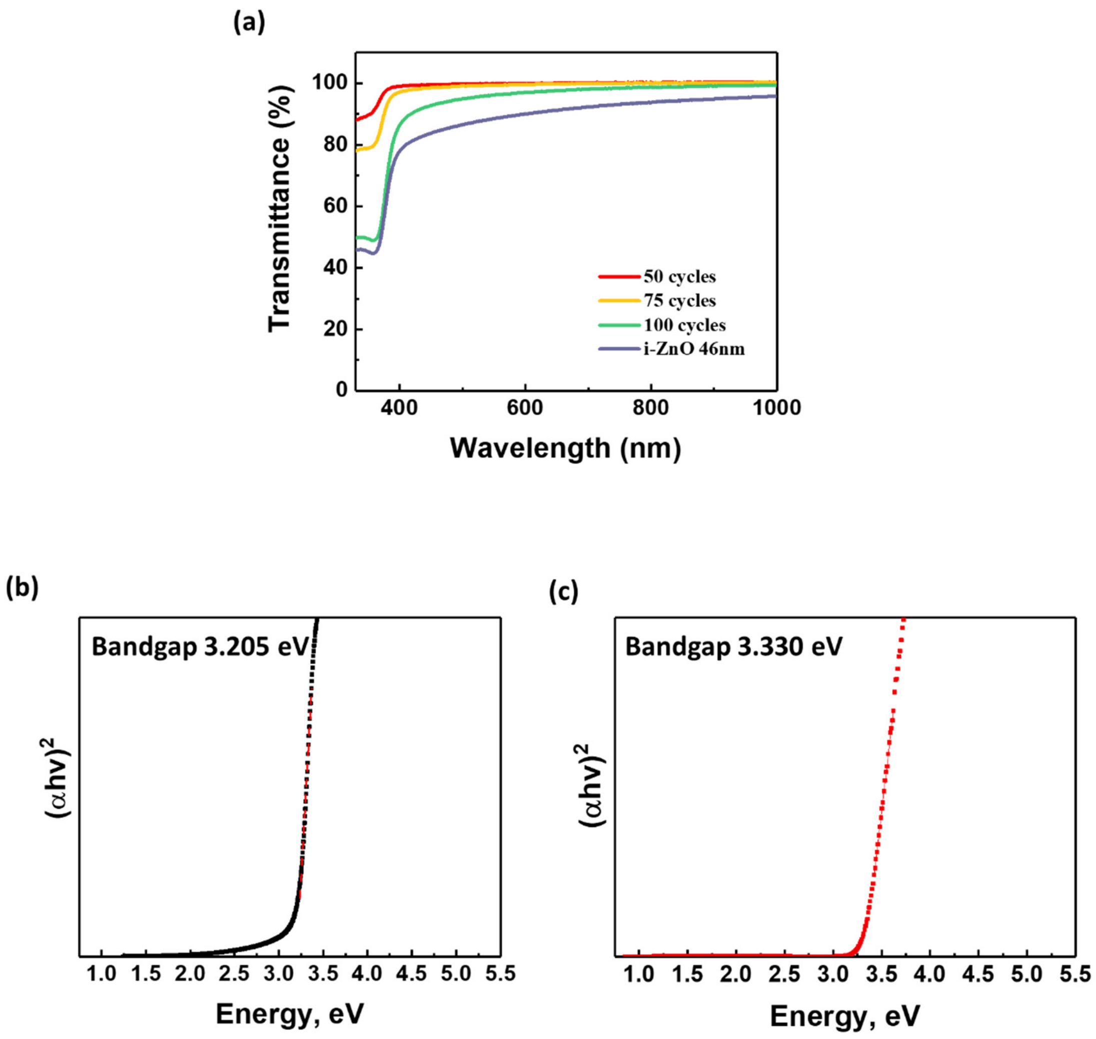

Figure S2. Transmittance and optical band gap properties of the i-ZnO and A-ZnO; (a) transmittance spectra of i-ZnO and A-ZnO, (b) optical band gap of i-ZnO, and (c) optical band gap of A-ZnO.
